# A case report and review of literature of Dieulafoy's disease of bronchus: A rare life-threatening pathologic vascular condition

**DOI:** 10.1097/MD.0000000000014471

**Published:** 2019-02-15

**Authors:** Pengcheng Zhou, Wei Yu, Kelin Chen, Xuelian Li, Qianming Xia

**Affiliations:** a Department of Respiratory Medicine, Hospital of Chengdu University of Traditional Chinese Medicine; b Clinical Medical School, Chengdu University of Traditional Chinese Medicine, Chengdu, Sichuan province, P.R. China.

**Keywords:** bronchial artery embolization, bronchoscopy, bronchus, Dieulafoy's disease, hemoptysis

## Abstract

**Rationale:**

Dieulafoy's lesions are characterized by the presence of a dysplastic artery in the submucosa, most frequently associated with gastrointestinal hemorrhage. They are rarely identified in the bronchial submucosa and can cause massive or fatal hemoptysis

**Patient concerns:**

The patient was a 62-year-old male farmer with intermittent hemoptysis of approximately 2 years duration and a definite diagnosis could not be established.

**Diagnosis:**

A thorax-computed tomography at our hospital revealed that the bronchus of left lower lobe was narrowed with associated local atelectasis, and lung cancer was suspected. A bronchoscopy showed a slit-like stenosis of the left lower lobe, swollen and smooth mucosa, and a significantly wider subsection carina.

**Interventions:**

A fatal hemorrhage occurred during biopsy and, rescue and resuscitation measures were immediately taken. A double-lumen endotracheal intubation was implanted and single-lung ventilation was started to maintain oxygenation. Hemoptysis completely stopped after bronchial artery embolization.

**Outcomes:**

The patient eventually died of disseminative intravascular coagulation and multiple organ failure. Bronchial arteriography and subsequent autopsy confirmed Dieulafoy's disease of the bronchus.

**Lessons:**

In cases with recurrent unexplained hemoptysis, where CT chest or thoracic radiography show no abnormalities, pulmonologist should suspect a bronchial Dieulafoy's disease and avoid blindly performing bronchoscopy guided biopsy, which may result in fatal hemoptysis.

## 1 Introduction

Dieulafoy's disease was first reported by the French doctor Georges Dieulafoy in 1898.^[1]^ It occurs most frequently in the gastrointestinal tract and is also called gastric submucosal aneurysm or Dieulafoy's ulcer.^[2–4]^ Dieulafoy's disease of bronchus is extremely rare, characterized by the presence of a dysplastic artery in the bronchial submucosa.^[5]^ Though the main symptom at presentation is hemoptysis or, occasionally, fatal hemoptysis in literature report,^[4]^ actually, this disease has various manifestations and it is not specific. It may be completely asymptomatic and diagnosed as an incidental founding on bronchoscopy.^[6,7]^ Therefore, it is still a big challenge to diagnose Dieulafoy's disease quickly and accurately. In order to improve the understanding of this disease for clinician, reduce misdiagnosis and underdiagnosis, we have presented this case report and literature review. The patient was a 62-year-old male with recurrent hemoptysis and cough, chest tomography shown left lower lobe atelectasis and lung cancer was suspected. Massive hemorrhage occurred immediately when bronchoscope-guided biopsy was attempted. The patient finally died despite immediate rescue measures and bronchial artery embolization. This reminds pulmonologists to pay more attention to this disease and avoid blindly performing bronchoscopy-guided biopsy, which may result in fatal hemoptysis.

## 2 Case presentation

**Figure 1:**
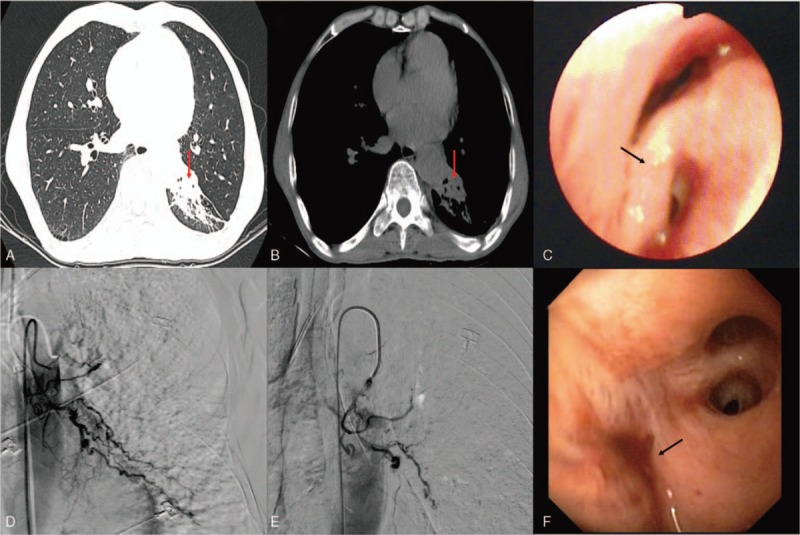
(A and B) Chest computed tomography showing bronchial stenosis in the left lower lobe, accompanied with local atelectasis (red arrow), emphysema, pulmonary bullae, and local thickened pleura. (C) Bronchoscopy showing a slit-like stenosis of the dorsal segment of left lower lobe, edematous, smooth mucosa, and widening of carina (black arrow), no abnormal vessels or active bleeding is seen. (D) Selective bronchial arteriogram showing a dilated, tortuous left lower lobe bronchial artery and profusely hypervascularized dorsal segment of left lower lobe. (E) Transcatheter embolization of the hypertrophic bronchial artery using poly-vinyl alcohol particles (PVA) 500 μm in diameter. After embolization, DSA reveals complete disappearance of the abnormal artery. (F) The opening of left lower bronchial lobe is occluded, but there is no active hemorrhage after BAE (black arrow).

The patient was a 62-year-old male farmer and was admitted to our hospital for intermittent hemoptysis of approximately 2 years duration. The patient had a smoking history of 360 pack-years for 30 years and was suffering from chronic obstructive pulmonary disease and pulmonary bullae. Before admission to our hospital, the patient had been diagnosed with and treated for pneumonia and bronchiectasis with antibiotics and hemostatics. At our hospital, the physical examination was normal, and a thorax computed tomography (CT; Fig. 1A and B) was performed, which revealed that the bronchus of left lower lobe was narrowed with associated local atelectasis, emphysema, pulmonary bullae, and thickened pleura. In order to confirm the diagnosis and exclude lung cancer, a bronchoscopy was performed, which showed a slit-like stenosis at the dorsal bronchial segment of the left lower lobe, swollen and smooth mucosa, and a significantly wider subsection carina. No abnormal vessels or active bleeding was noted and the other bronchus was normal (Fig. 1C). A large hemorrhage occurred immediately when biopsy was attempted at the carina of dorsal bronchial segment. The entire left airway immediately filled with blood, and rescue and resuscitation measures (e.g., ipsilateral position, increased oxygen flow, thrombin airway instillation, intravenous infusion with vasopressin, and sustained aspiration) were immediately taken. The patient developed hemorrhagic shock, and anti-shock measures were implemented (e.g., intravenous infusion with polygeline, compound sodium chloride solution, dopamine, and type-A erythrocyte suspension). Finally, the bleeding stopped 20 minutes later, with an estimated loss of 1500 ml. The patient was subsequently referred to the intensive care unit for further observation. Dieulafoy's disease of bronchus was suspected and bronchial arteriography was recommended after a multidisciplinary discussion, but the patient's family members refused to give permission for the same. The patient developed a recurrent massive hemoptysis 3 hours later; double-lumen endotracheal intubation was performed immediately and single-lung ventilation was started to maintain oxygenation. A bronchial arteriography was performed subsequently with the consent of patient's family, which showed abundant distorted and hyperplastic bronchial arteries in the left lower lobe, accompanied with contrast agent overflow from the vascular lesion (Fig. 1D). Finally, the aneurysmal vessel was embolized with poly-vinyl alcohol (PVA) particles of 500 μm diameter and hemoptysis stopped completely (Figs. 1E and F). After the operation, the patient was transferred to intensive care. Unfortunately, the patient eventually died of disseminative intravascular coagulation and multiple organ failure. Bronchial arteriography and subsequent autopsy confirmed Dieulafoy's disease of the bronchus.

## 3 Discussion

Dieulafoy's disease was first reported by the French doctor Dieulafoy in 1889,^[1]^ also called gastric submucosal aneurysm or Dieulafoy's ulcer, is characterized by the presence of a dysplastic artery in the submucosa, usually in gastrointestinal tract. However, bronchial Dieulafoy's disease is extremely rare, and was reported for the first time in 1995 by Sweerts.^[1]^ The hyperplastic bronchial arteries usually travel through the superficial mucosa of the bronchus or are exposed within the lumen. We performed a comprehensive analysis of the literature from 1 January 1995 to 1 August 2018 on the Medline (National Library of Medicine, USA), Cochrane Library (UK), CNKI(China) and SinoMed (China) databases using the following keywords

Dieulafoy's disease, Dieulafoy's lesion, and Dieulafoy's disease of the bronchus. The results were restricted to articles available in English and/or Chinese. We retrieved 24 English articles ^[1–24]^ and 19 Chinese articles,^[25–43]^ which reported 72 cases (73, after including the present case). (Tables 1 and 2)

**Table 1:**
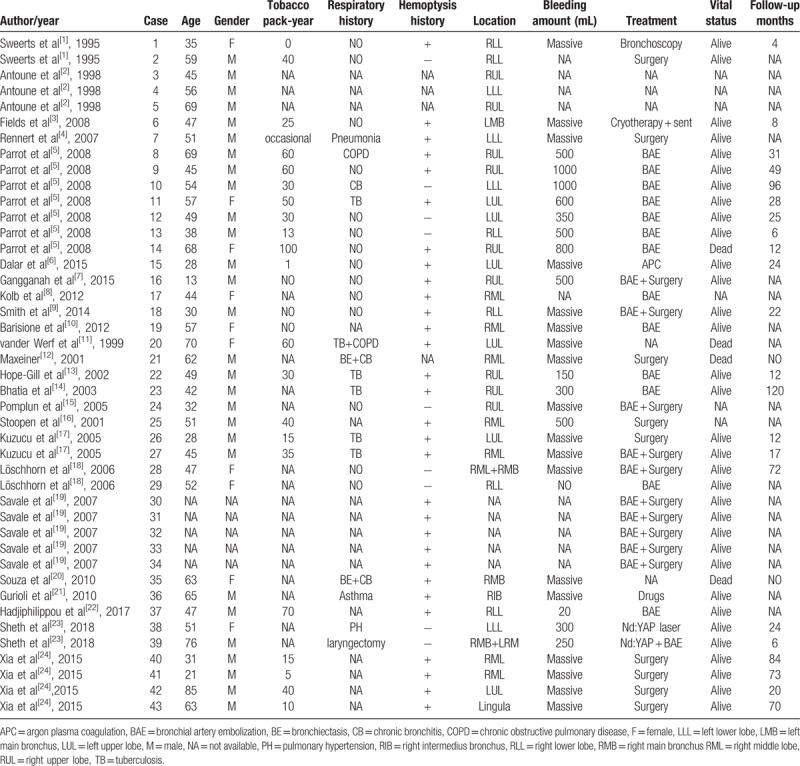
Clinical manifestations of Dieulafoy's disease of bronchus (literatures reported in English).

**Table 2:**
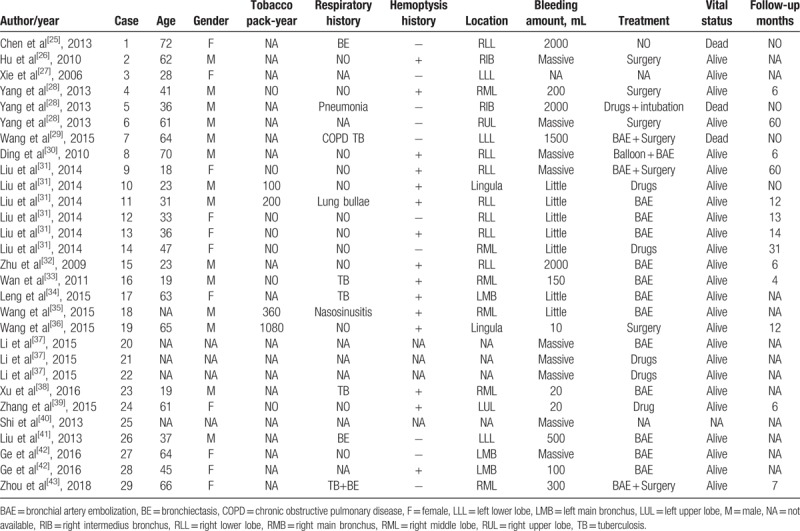
Clinical manifestations of Dieulafoy's disease of bronchus (literatures reported in Chinese).

Bronchial Dieulafoy's lesion presented nonspecific symptoms, including a burst of cough, recurrent hemoptysis, shortness of breath, chest discomfort, etc. From our literature review, 61% patients had a previous history of hemoptysis (45/73), 20% patients had cough (15/73), 6% had dyspnea (5/73), and 2% had chest pain (2/73). All the patients were admitted to hospital because of coughing blood (73/73). The patient we report presented with intermittent hemoptysis about 2 years’ duration, and had been misdiagnosed with pneumonia and bronchiectasis in the past. Therefore, Dieulafoy's disease of the bronchus should be considered in patients suffering from recurrent, unexplained, or massive hemoptysis.

Bronchial Dieulafoy's disease presents no typical findings on CT thorax. From our literature review, 25% patients had ground-glass opacity (18/73), 20% patients had pulmonary infection (15/73), 7% patients had bronchiectasis (5/73), 5% patients had lump (4/73), 5% patients had atelectasis (4/73), 4% patients had lung texture disorder and emphysema (3/73), 3% patients had nodules (2/73), 1% patients showed a soft tissue in bronchus (1/73), 1% patients had pleural effusion (1/73), and 32% patients had no obvious abnormalities (23/73). The CT findings of this case in our article were not consistent with the literature reported, it showed bronchial stenosis, local atelectasis, and thickened pleura of left lower lobe, which was extremely difficult to distinguish from lung cancer. Therefore, it was difficult to diagnose Dieulafoy's disease of bronchus by CT alone.

The right bronchial has been involved most frequently; Yang et al^[28]^ summarized the lesion sites for 22 cases of Dieulafoy's disease of the bronchus, which revealed 16 cases occurred in right bronchus, only 4 cases occurred in left. In our literature review, 58% cases were situated in right bronchus (42/73), 29% in the left bronchus (21/73), and 1% in bilateral bronchi (1/73); in 12% cases, the lesion site was unknown (9/73). Dieulafoy's disease remains a condition of uncertain etiology, and it is unclear that why it favors the right bronchus. Pomplun et al^[15]^ postulated that chronic inflammation injury of bronchi and congenital vascular malformation were the main causes. Stoopen et al^[16]^ also suggested a congenital origin. Most patients reported in the literature had a history of smoking and respiratory disease, especially tuberculosis, chronic obstructive pulmonary disease, pneumonia, bronchiectasis, and chronic bronchitis; all these might be potential risk factors contributing to bronchial Dieulafoy's disease.

Bronchial Dieulafoy's disease is usually seen as a nodular protrusion into the lumen on bronchoscopy: the superficial mucosa is smooth, the diameter and height always < 5 mm, the color usually normal or mildly hyperemic, with or without pulsatility, and sometimes with a white cap. Occasionally, the malformed vessels can be directly observed in white light bronchoscopy, appearing as a distorted earthworm or root.^[4,5]^ In our literature review, 68% cases showed a nodule (50/73), 5% cases had a lesion resembling a distorted earthworm (4/73), 4% cases showed soft tissue in the lumen (3/73), 3% cases presented with local blood clots (2/73), 1% case appeared like a distorted root with pulsation (1/73), and 1% showed mucosal ulcers with vascular pulsation (1/73); 16% cases had no abnormalities (12/73). The case reported in our article showed a slit-like stenosis at the dorsal bronchial segment of the left lower lobe, swollen and smooth mucosa, and a significantly wider subsection carina. No abnormal vessel or active bleeding was noted and the other bronchus was normal. All the manifestations were not consistent with the literature reported.

Angiography is a useful tool to diagnose Dieulafoy's disease of the bronchus; it shows abundant hypervascularized peri-lesional vessels; these vessels under the superficial bronchial mucosa can be dilated, tortuous, or abnormally hyperplastic.^[4,5,28]^ Most of these vessels originate from bronchial artery, and only a few from pulmonary artery. In our literature review, 97% cases originated from bronchial artery (71/73), only 3% cases originated from the pulmonary artery (2/73). The case we reported was also diagnosed by angiography.

The histopathological examination shows typical findings; mostly showing a superficial, dysplastic, tortuous, and dilated bronchial artery under the bronchial epithelium, projecting or directly communicating with the lumen.^[5,18,30]^

In cases with recurrent unexplained hemoptysis, where CT chest or thoracic radiography shows no abnormalities, it is necessary to take bronchial Dieulafoy's disease into consideration. The diagnosis depends on a combination of clinical manifestations, CT chest, bronchoscopy, pulmonary angiography, and even pathology. Angiography and biopsy are confirmatory diagnostic investigations. It is usually difficult to discern submucosal vascular lesions on conventional white light bronchoscopy. Some researchers consider that endobronchial ultrasonography (EBUS)^[7,21]^ or narrow-band imaging (NBI)^[36]^ is more useful in diagnosing bronchial Dieulafoy's disease; these can detect submucosal vasculature, avoid blind biopsy, and prevent fatal hemoptysis. The present case was diagnosed by pulmonary arteriography.

Once the diagnosis of bronchial Dieulafoy's disease is confirmed, bronchial artery embolization or lobectomy should be performed in time, but the success rate of bronchial artery embolization is only 40%.^[18]^ For patients with recurrent hemoptysis, surgical resection is recommended. In our literature review, 55% patients had undergone successful bronchial arterial embolization (40/73), 45% underwent surgical resection (33/73), and 33% cases had undergone both procedures (24/73). Nevertheless, 11% patients died due to fatal airway hemorrhage (8/73). The drug has limited effect to stop bleeding and patients often die of asphyxia when fatal hemoptysis occurs. Therefore, an endotracheal intubation and mechanical ventilation performed immediately is the key point. Single-lumen endotracheal intubation is easy to perform and maintain oxygenation, but it cannot block the bleeding on the affected side. We have solved the above problems by using a double lumen endotracheal intubation and gained valuable time for follow-up treatment. However, we need to emphasize that double-lumen endotracheal intubation is difficult to implant, which requires skilled technology and good teamwork and reasonable rescue measures need to be combined.

Accompanied with the development of medical technology, interventional therapy with bronchoscope can play an important role here. Fields et al^[3]^ had successfully administered cryotherapy and implanted a Dumon silicone stent to cure a case of Dieulafoy's disease with recurrent hemoptysis. Dalar et al^[6]^ had successfully used argon plasma coagulation (APC) to treat a case of bronchial Dieulafoy's disease, with no recurrence of hemoptysis during follow-up. Hadjiphilippou et al^[2]^ successfully ablated a bronchial Dieulafoy's lesion with bronchoscopic Nd: YAG laser in two patients and concluded that the procedure can provide durable relief from recurrent hemoptysis, but it should only be attempted by an experienced physician.

## 4 Conclusions

Despite the literature report of bronchial Dieulafoy disease is increasing, clinician still need to pay more attention to it. It is crucial to be aware of the abnormality of the vasculature while performing bronchoscopy, because biopsy may result in massive hemorrhage, with a possible fatal outcome. It is important that researches are conducted for simple, effective, and specific methods to diagnose this disease.

## Acknowledgments

We thank all members of the Department of Thoracic Surgery, the Department of Vascular surgery, the Department of Anesthesiology, and the ICU of the affiliated hospital of Chengdu University of Traditional Chinese Medicine in the management of this patient.

## Author contributions

(I) Conception and design: Pengcheng Zhou

(II) Administrative support: Qianming Xia

(III) Provision of study materials or patients: Kelin Chen

(IV) Collection and assembly of data: Wei Yu

(V) Data analysis and interpretation: Xuelian Li

(VI) Manuscript writing: All authors

(VII) Final approval of manuscript: All authors

**Conceptualization:** Pengcheng Zhou.

**Data curation:** Wei Yu.

**Formal analysis:** Xuelian Li.

**Investigation:** Xuelian Li.

**Methodology:** Qianming Xia.

**Project administration:** Qianming Xia.

**Resources:** Kelin Chen.

**Writing – original draft:** Pengcheng Zhou, Wei Yu, Kelin Chen, Xuelian Li, Qianming Xia.

**Writing – review & editing:** Pengcheng Zhou.
